# Citrate-buffered Yamanaka medium allows to produce high-yield bacterial nanocellulose in static culture using *Komagataeibacter* strains isolated from apple cider vinegar

**DOI:** 10.3389/fbioe.2024.1375984

**Published:** 2024-05-15

**Authors:** Dariela Núñez, Patricio Oyarzún, Rodrigo Cáceres, Elizabeth Elgueta, Maribet Gamboa

**Affiliations:** ^1^ Departamento de Química Ambiental, Facultad de Ciencias, Universidad Católica de la Santísima Concepción, Concepción, Chile; ^2^ Centro de Investigación en Biodiversidad y Ambientes Sustentables (CIBAS), Universidad Católica de la Santísima Concepción, Concepción, Chile; ^3^ Facultad de Ingeniería, Arquitectura y Diseño, Universidad San Sebastián, Concepción, Chile; ^4^ Departamento de Ecología, Facultad de Ciencias, Universidad Católica de la Santísima Concepción, Concepción, Chile

**Keywords:** bacterial nanocellulose, Komagataeibacter, citrate buffer, apple vinegar, pH control, isolated strains

## Abstract

Bacterial nanocellulose (BNC) is a sustainable, renewable, and eco-friendly nanomaterial, which has gained great attentions in both academic and industrial fields. Two bacterial nanocellulose-producing strains (CVV and CVN) were isolated from apple vinegar sources, presenting high 16S rRNA gene sequence similarities (96%–98%) with *Komagataeibacter* species. The biofilm was characterized by scanning electron microscopy (SEM), revealing the presence of rod-shaped bacteria intricately embedded in the polymeric matrix composed of nanofibers of bacterial nanocellulose. FTIR spectrum and XRD pattern additionally confirmed the characteristic chemical structure associated with this material. The yields and productivities achieved during 10 days of fermentation were compared with *Komagataeibacter xylinus* ATCC 53524, resulting in low levels of BNC production. However, a remarkable increase in the BNC yield was achieved for CVV (690% increase) and CVN (750% increase) strains at day 6 of the fermentation upon adding 22 mM citrate buffer into the medium. This effect is mainly attributed to the buffering capacity of the modified Yakamana medium, which allowed to maintain pH close to 4.0 until day 6, though in combination with additional factors including stimulation of the gluconeogenesis pathway and citrate assimilation as a carbon source. In addition, the productivities determined for both isolated strains (0.850 and 0.917 g L^−1^ d^−1^) compare favorably to previous works, supporting current efforts to improve fermentation performance in static cultures and the feasibility of scaling-up BNC production in these systems.

## Highlights

Isolated *Komagataeibacter* strains enables top-performing yields and productivities of BNC in modified citrate-buffered Yamanaka medium.
Bacterial nanocellulose was characterized and chemically confirmed by FTIR, XRD and SEM techniques.

## 1 Introduction

Advanced nano-biomaterials are paramount to help catalyze the transition from a petrochemical to a bio-based and green economy, as they can be produced from renewable sources and converted into a variety of high-value-added products (Bennich and Belyazid, 2017; Guzman-Puyol et al., 2022). Nanocellulose is currently driving innovation in a variety of fields thanks to its unique structure, extraordinary attributes and biological properties (Qi et al., 2023). Despite nanocellulose can be obtained from lignocellulosic biomass via mechanical or chemical treatments (Qi et al., 2023), these processes tend to be energy-intensive and pose environmental hazards due to the use and emission of toxic chemicals (Cacicedo et al., 2016). By contrast, bacterial nanocellulose (BNC) has been reported as an inexhaustible, renewable material and the highest hallmark for green materials (Thomas et al., 2018). BNC is additionally produced in a pure form (≥98%), without the presence of lignin, pectin, hemicellulose, and other biogenic products that are normally associated with plant-based cellulose.

BNC is an exopolysaccharide that forms a three-dimensional reticulated network composed of nanofibers with diameters ranging from 20 to 100 nm (Puri, 1984). This nanomaterial is produced by bacteria from a wide variety of genera, which currently include *Komagataeibacter* (former *Gluconacetobacter*), *Aerobacter*, *Rhizobium*, *Sarcina*, *Azotobacter*, *Agrobacterium*, *Pseudomonas*, *Alcaligenes,* among others (Chawla et al., 2009; Islam et al., 2017). However, several *Komagataeibacter* strains were recently reclassified based on genomic studies as members of a novel genus termed *Novacetimonas gen. nov* (Brandão et al., 2022; Neelima et al., 2023). *Komagataeibacter* species are well-recognized BNC producers, which are Gram-negative, rod-shaped, and obligate aerobic bacteria. In particular, *K. xylinus* is considered as the model organism in genetic and biochemical investigations of cellulose biosynthesis, having the ability to produce the highest levels of BNC from a wide range of carbon and nitrogen sources (Chawla et al., 2009).

BNC possess outstanding mechanical properties and water holding capability associated with its high porosity, high tensile strength, high specific surface area, high aspect ratio (Cacicedo et al., 2016), high degree of polymerization (DP) and high degree of crystallinity (up to 84%–89%), which together with excellent biocompatibility and biodegradability make BNC of great interest for applications in biomedicine and various technology fields (Petersen and Gatenholm, 2011; Picheth et al., 2017). Accordingly, this material has been successfully applied in bioelectronics (Zhang et al., 2021), environmental applications (Núñez et al., 2020), food industry and a long number of biomedical and tissue engineering applications, such as wound dressing (Portela et al., 2019), bone tissue scaffolds (Torgbo and Sukyai, 2018), artificial skin (Liu et al., 2022), vascular and cartilage implants (Petersen and Gatenholm, 2011; Lee et al., 2014; Mohite and Patil, 2014; Cacicedo et al., 2016; Picheth et al., 2017). However, the widespread application of BNC is hindered due to its low yield and low productivity, the instability of microbial strains and cost limitations that currently interfere with its large-scale mass production. Accordingly, the isolation of highly productive strains has become a prominent issue, especially because *Komagataeibacter* spp. show significant inter and intra species variability in BNC production (Gullo et al., 2017; Ryngajłło et al., 2020; Brugnoli et al., 2021; La China et al., 2021; Anguluri et al., 2022).

BNC production is directly affected by the cultivation conditions, being the pH a critical parameter that needs to be controlled in the medium to prevent pH from dropping below 4 and to cause a decrease in carbon assimilation and BNC production (Klemm et al., 2001). *Komagataeibacter* spp. typically causes a decrease in the pH during first 3–4 days due to the accumulation of organic acids (i.e., gluconic and acetic acids) (Esa et al., 2014; Blanco Parte et al., 2020; Lahiri et al., 2021). A slightly acidic pH is typically associated with maximum BNC production, however; optimum pH for BNC yield is a strain-dependent trait (Dirisu et al., 2017; Fernandes et al., 2020).

In this study, we investigated the production capability of nanocellulose with two novel bacterial strains isolated from apple vinegar, which were identified as belonging to the *Komagataiebacter* genus. The yield and productivities of BNC were compared with *K. xylinus* ATCC 53524, proving the use of citrate buffer in Yamanaka medium provides a straightforward method to keep de pH controlled and to achieve top-performing production. These results are of interest in the light of current efforts to select novel bacterial strain, as well as low-cost approaches to improve fermentation performance and the feasibility of scaling-up static culture systems.

## 2 Materials and methods

### 2.1 Screening and isolation of bacterial strains

Two bacterial strains were isolated from apple cider vinegar samples obtained from a local market in Concepción, Chile. For isolating the strains, 0.5 mL of vinegar sample were streaked onto Hestrin–Schramm (HS) agar plate containing 2.0% D-glucose, 0.5% peptone, 0.5% yeast extract, 0.27% Na_2_HPO_4_ and 0.115% citric acid, and incubated at 28°C for 6 days (Hestrin and Schramm, 1954). The screening procedure was based on the identification of white-cream colonies with round, bigger and mucous structure, which is indicative of the presence of BNC-producing bacteria. To confirm the presence of positive bacteria, the grown colonies were streaked out to HS liquid medium and cultured for 6 days in 125 mL Erlenmeyer flasks under static conditions at 28°C. Positive results were confirmed by the formation of a pellicle at the air–liquid interface of the medium and subsequent characterization by Fourier transform infrared (FTIR) after pellicle purification. BNC-producing isolates were preserved in vials containing a 20% glycerol solution and stored at −80 °C. Another strain employed in this study was sourced from the American Type Culture Collection (*K. xylinus* ATCC 53524).

### 2.2 Bacterial identification

The cellulose-producing bacteria were identified by 16S rRNA gene sequencing analysis performed at Macrogen Inc. (Daejeon, South Korea). Genomic DNA of the isolated strains was extracted using Wizard^®^ Genomic DNA Purification Kit (Promega Corporation) and subsequently amplified using a Heal force Thermal Cycler (model T960). The thermal cycler was programmed with an initial denaturation step lasting 5 min at 94°C. Subsequently, 30 cycles were performed, consisting of 1 min at 94°C for additional denaturation, 1 min at 55°C for annealing, and 2 min at 72°C for extension. Finally, a concluding extension step was carried out at 72°C for 10 min.

PCR amplification of the 16S rDNA was performed using primer sets 27f (5′-AGA​GTT​TGA​TCC​TGG​CTC​AG-3′) and 1492r; (5′-ACG​GCT​ACC​TTG​TTA​CGA​CTT-3′) which amplify the nearly full-length of 16S rDNA.

### 2.3 Sequence data analysis

The quality of the sequences was checked using CodonCode Aligner version 1.2.4 software (https://www.codoncode.com/aligner/). No ambiguous bases were detected, and the low-quality bases were removed from the start and end of the sequences. The nucleotide sequences were compared with publicly available 16S rRNA gene reference sequences in the GenBank databases using BLASTN (https://blast.ncbi.nlm.nih.gov/Blast.cgi). The nucleotide sequence was compared with those available sequences on the National Center for Biotechnology Information GenBank (NCBI GenBank) database employing BLASTn through the Basic Local Alignment Search Tool (BLAST). Homologous sequences were retrieved, and multiple alignments were executed through the MAFFT alignment online program with default settings (https://mafft.cbrc.jp/alignment/server/). The phylogenetic tree was constructed using a maximum likelihood approach in PhyML v3.0 (Guindon and Gascuel, 2003) under a GTR model of evolution as determined by Modeltest v3.7 (Posada and Crandall, 1998) and bootstrapping of 1,000 replications. Uncorrected pairwise p–distances among individuals were generated with ape package (Paradis and Schliep, 2019) in R v3.3 (R Core Team, https://www.r-project.org/).

### 2.4 Static culture using Yamanaka and modified Yamanaka media

To prepare the inoculum of the strains (two isolated and one reference strain), agar plates (containing HS medium with 3.75 g L^−1^ of agar) were initially streaked with glycerol stocks of each strain and then incubated for 3 days at 30°C. Subsequently, a single colony from each agar plate was transferred into a 50 mL flask containing 10 mL of HS basal medium and allowed to incubate for 72 h on an orbital shaker at 150 rpm and 28°C. The resulting cell suspension served as the inoculum for subsequent experiments.

Kinetic studies investigating glucose consumption and cellulose production were conducted to evaluate the impact of incorporating citrate buffer into Yamanaka media. The experiments were performed under static culture conditions, at 28°C for 2, 4, 6, 8, and 10 days in duplicate setups. Petri dishes containing 40 mL of Yamanaka medium (comprising 2% glucose, 0.5% ammonium sulfate, 0.5% yeast extract, 0.3% K_2_HPO_4_, and 0.005% magnesium sulfate) were inoculated with 500 µL of the previously prepared cell suspension. Identical experiments were set up using Yamanaka medium supplemented with 22 mM citrate buffer (3.74 g L^−1^ of sodium citrate and 1.35 g L^−1^ of citric acid, pH 5.0), referred to hereafter as modified Yamanaka medium. Bacterial cultures using Yamanaka and modified Yamanaka medium were harvested at 2, 4, 6, 8 and 10 days for pH measurements, cellulose, and glucose quantification. In addition, BNC pellicles obtained at 10 days of fermentation were purified according to sub-section 2.5 and freeze-dried for subsequent FTIR and XRD analyses. The morphology of BNC pellicles (purified and lyophilized) produced with Yamanaka medium and citrate-buffered Yamanaka medium was recorded using a digital camera. For lyophilization, samples were frozen overnight (at −20°C) and then dehydrated using a freeze dryer (Operon, FDU-7012) for 72 h at −70°C.

### 2.5 Quantification of cellulose

The BNC yield (in g L^−1^) was quantified by dry weight. After each fermentation period (2, 4, 6, 8 and 10 days), the formed pellicle in the petri-dish culture was harvested and centrifuged at 4,500 rpm to remove culture broth. The pellet was washed-centrifuged three times with distilled water to remove any residual content of the medium, while the remaining culture broth was collected for pH and glucose analyses. For further purification and bacterial removal, the resulting pellet was suspended in 100 mL of NaOH 0.1 M and maintained at 80°C for 3 h (Ruka et al., 2012; Ye et al., 2019). Upon cell lysis, the samples were centrifuged and washed three times with distilled water. The BNC obtained was dried at 80°C for 3 days and its weight (in g) was divided by the total culture volume (L) to obtain the BNC yield (in g L^−1^) at 2, 4, 6, 8 and 10 days.

### 2.6 Quantification of glucose

The glucose concentration (g L^−1^) in the culture broth samples (after removing the cellulose pellicle) was determined by the 3,5-dinitrosalicylic acid (DNS) method to quantify total reducing sugars. To perform the assays, 500 µL of diluted samples (1:10) of the culture broth were placed in 15 mL test tubes, followed by the addition of 1 mL of DNS reagent. The reaction mixture was heated to 100 °C in a heating block for 5 min. Subsequently, 5 mL of distilled water were added. A calibration curve with glucose (0.2–1.0 g L^−1^) was prepared following same procedure described above. The absorbance spectra of the test samples and calibration curve were measured at 540 nm using quartz cuvettes with a 2 mm path length in a UV–vis spectrophotometer (Spectronic, 20 Genesys).

### 2.7 Material characterization

#### 2.7.1 Scanning electron microscopy (SEM)

Bacterial morphology and microstructural features of the BNC biofilms and BNC purified biofilms produced by the three strains under study (CVV, CVN and reference strain), were characterized by scanning electron microscopy (SEM) using two different microscopes Autoscan (ETEC) and Vega3 Easyprobe SBU (TESCAN). Prior to the analysis, the samples were fixed in 2% glutaraldehyde for 2 h, subsequently dehydrated in alcoholic series and then in a critical point dryer (EM CDP 300, Leica Microsystem). Finally, the samples were sputter-coated with gold before examination in the microscope (EM ACE200, Leica Microsystem).

#### 2.7.2 Fourier transform infrared spectroscopy (FTIR)

The FTIR analysis of freeze-dried BNC samples was conducted using a Spectrum Two instrument from Perkin Elmer, which was equipped with a diamond attenuated total reflection (ATR) accessory. The samples were securely mounted using a high-pressure clamp, and the spectra were obtained by averaging 10 scans within the range of 400–4,000 cm^−1^ at a resolution of 4 cm^−1^.

#### 2.7.3 X-ray diffraction (XRD)

The freeze-dried BNC samples were placed evenly in a Lucite sample holder, and gently pressed by hand to form a compact layer. The analysis was carried out in a Bruker D8 Advance diffractometer at 40 kV and 40 mA, using Cu Kα radiation. The diffractogram was recorded in the range of 10°–30° with a step size 0.02° and step time of 1 s.

## 3 Results and discussions

### 3.1 Phylogenetic affiliation of strains CVV and CVN

The genotypic characterization of the isolated strains was performed by amplifying the 16S rDNA, resulting in fragments approximately 1,000 bp in size. The GenBank accession numbers for CVV and CVN 16S rRNA gene nucleotide sequences are PP194767 and PP195241, respectively.

**FIGURE 1:**
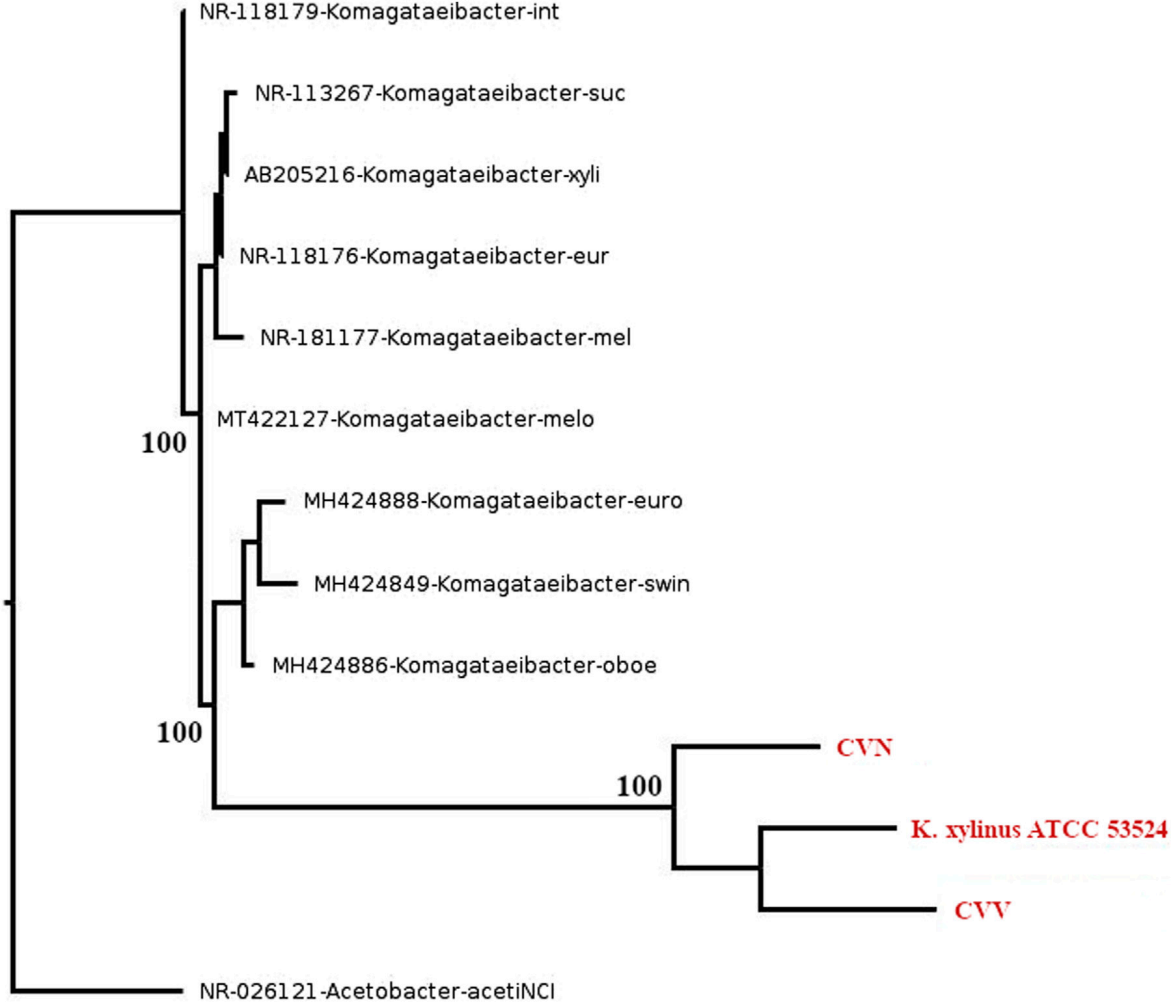
Phylogenetic reconstruction of 16S rRNA. The new isolated vinegar strains (CVN and CVV) and the reference strain *K. xylinus* ATCC 53524 are highlighted in red. The trees were constructed using the maximum likelihood approach. Bootstrap values are indicated at branching points (1,000 replicates). GenBank access number are provided before species names.

The 16S rRNA gene sequencing confirmed the affiliation of the two vinegar strains (CVN and CVV) to the *Komagataeibacter* genus since a similarity >94.5% (Yarza et al., 2014) was obtained when comparing 16S rRNA gene sequences of the isolated with sequences of other *Komagataeibacter* spp. The phylogenetic reconstruction of 16S rRNA is presented in Figure 1. The gene sequence of the strain CVV showed the highest similarity (97%–98%) with *K. xylinus* and *K. europaeus* strains (e-value score of 0.0). The strain CVN is potentially a new species, as sequence similarity of the 16S rRNA gen with respect to the most closely related species (96%) is lower than the cut-off value (98.7%) currently applied to delineate this taxonomic level (Yarza et al., 2014).

### 3.2 Material characterization

**FIGURE 2:**
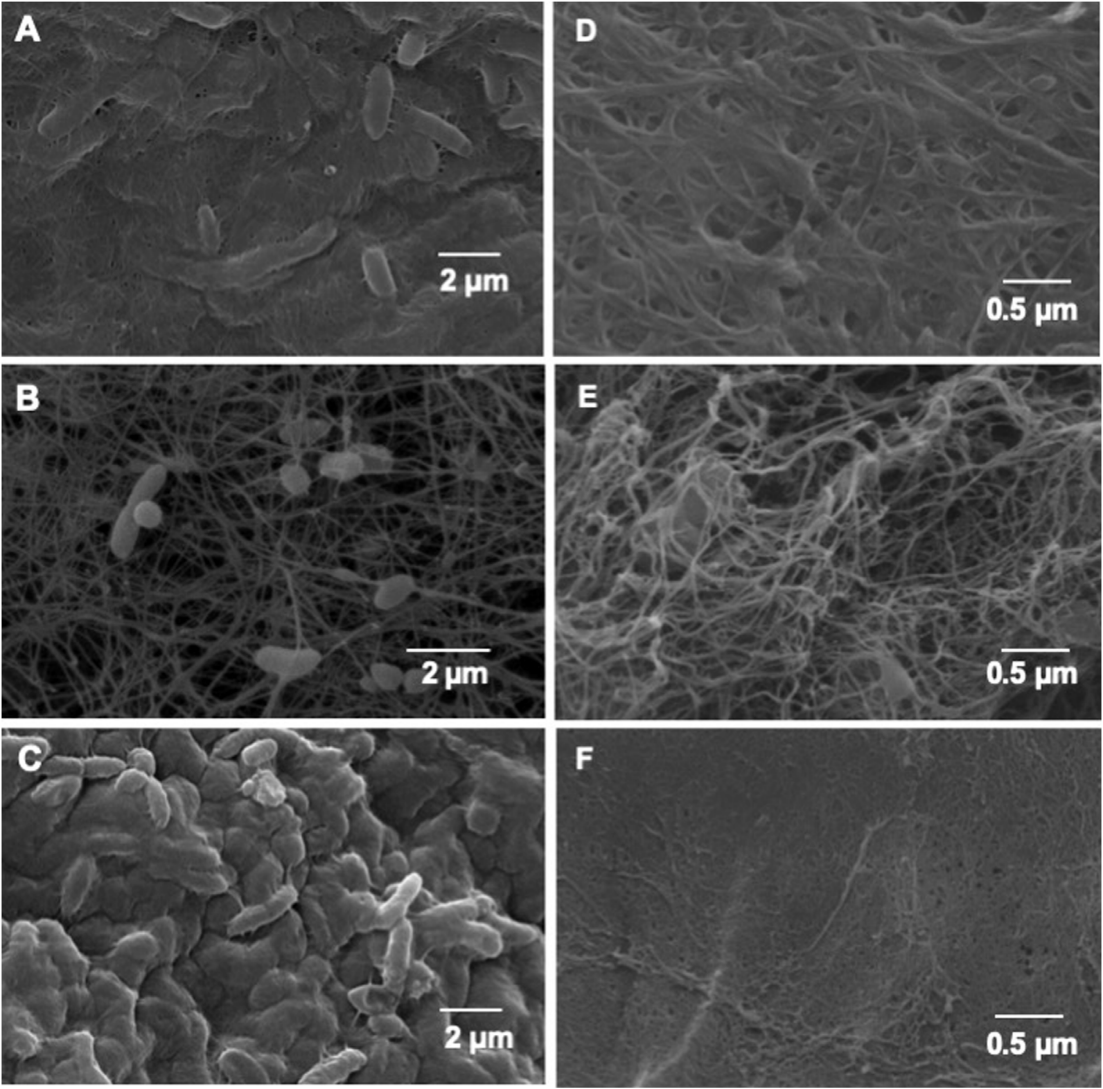
SEM micrographs of the BNC biofilm (left column) and of BNC purified from the biofilms (right column), produced by the Komagataeibacter strains. **(A)** BNC biofilm produced by *K. xylinus* ATCC 53524; **(B)** BNC biofilm produced by CVV strain; **(C)** BNC biofilm produced by CVN; **(D)** Purified BNC obtained from the biofilm formed by *K. xylinus* ATCC 53524; **(E)** Purified BNC obtained from the biofilm formed by CVV; **(F)** Purified BNC obtained from the biofilm formed by CVN.

**FIGURE 3:**
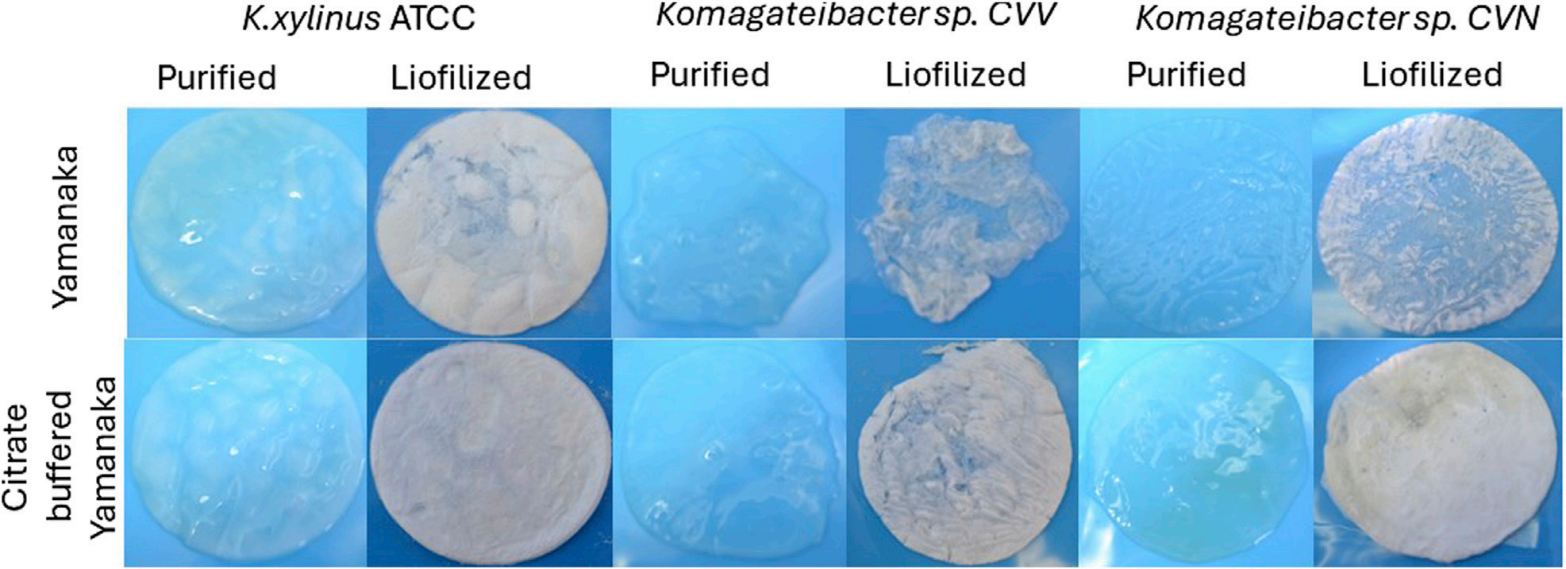
Photographs of the purified and lyophilized BNC pellicles obtained after 10 days of fermentation using Yamanaka and citrate-buffered Yamanaka medium.

Figures 2A–C present SEM micrographs of the BNC biofilm, which confirms that cellulose fibrils embedded in an exopolysaccharide matrix are secreted from the surface of rod-shaped cells. The three bacteria are similar size, showing higher abundancy in the biofilm produced by *K. xylinus* ATCC 53524 (A) and the biofilm produced by CVN (C) as compared with the biofilm produced by CVV strain (B). After purification with NaOH 0.1 N, BNC pellicles showed different morphologies, BNC produced by *K. xylinus* ATCC 53524 show higher porosity with an approximated diameter of 0.1–0.4 μm formed by thicker fibers (D), while BNC produced by CVV strain has a more loosed nanostructure, and BNC produced by the CVN strain show a more homogeneous structure with thinner pores of <0.1 μm. Macroscopic observation of purified and lyophilized BNC pellicles (Figure 3) reveals that *K. xylinus* ATCC 53524 and the CVN strain produced a dense structure when cultivated in standard and modified Yamanaka medium after 10 days of fermentation. However, the structural integrity of the BNC pellicles produced by CVV strain becomes compromised after purification and lyophilization using both media, consistently with the loosed structure observed in the SEM micrograph (Figure 2E).

**FIGURE 4:**
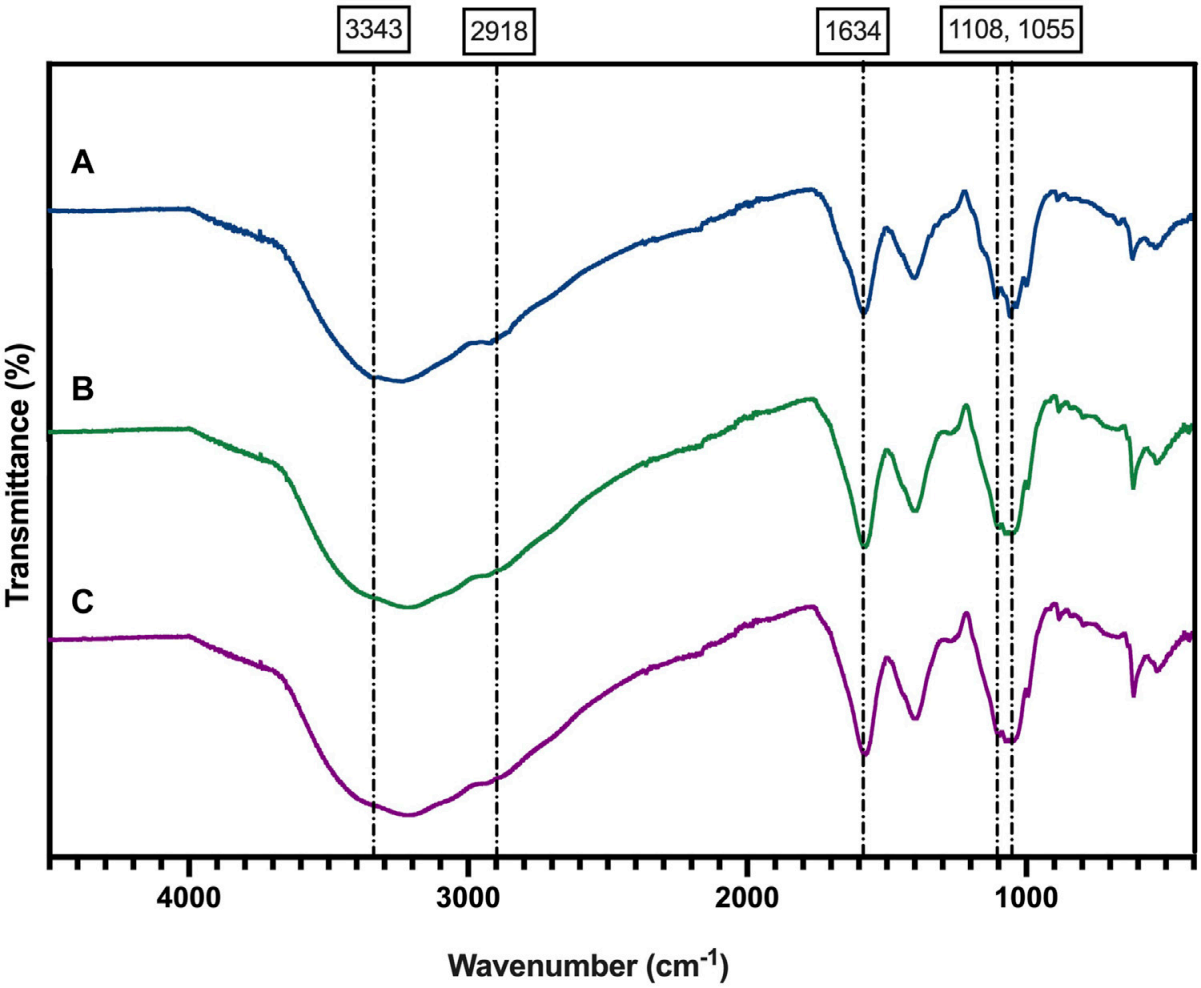
FTIR analysis of the BNC produced by the reference strain (*K. xylinus* ATCC 53524) **(A)**, isolated CVV strain **(B)**, and CVN strain **(C)**.

The ATR-FTIR spectrum of BNC (Figure 4) revealed the typical pattern described for this material (Kuo et al., 2016), including a broad band at 3,343 cm^−1^ that corresponds to the OH^−^ stretching vibrational mode. The peaks located at 2,918 cm^−1^ and at 1,634 cm^−1^ are attributed to C-H stretching and to the bending mode of water molecule (H-O-H), respectively (Salari et al., 2019). The peaks located at 1,360, 1,335 and 1,314 cm^−1^ are associated with CH_3_ deformation, OH deformation and CH bending vibrational modes of BNC (Wahid et al., 2019). The peaks found at 1,160 and 1,108 cm^−1^ correspond to C-O-C and 1,055 cm^−1^ to C-C, C-OH, C-H ring (Wahid et al., 2019).

**FIGURE 5:**
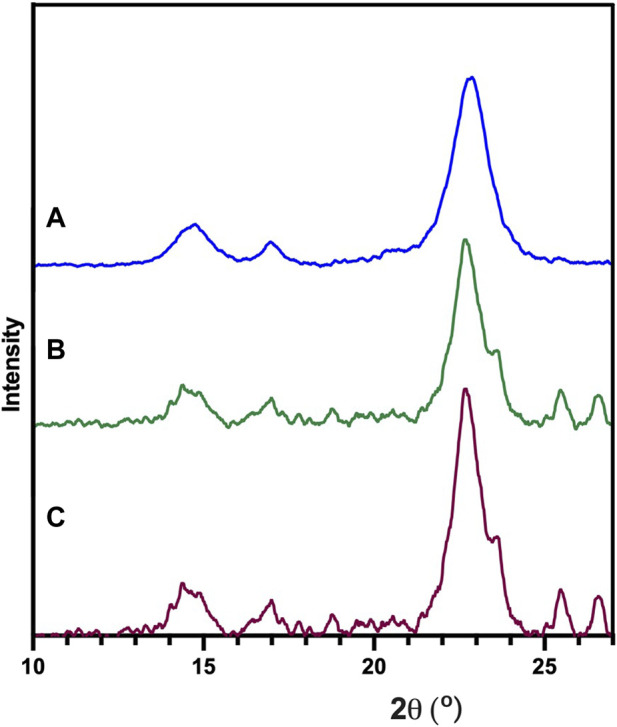
XRD analysis of the BNC produced by the reference strain (*K. xylinus* ATCC 53524) **(A)**, isolated CVV strain **(B)** and CVN strain **(C)**.

The structure of BNC was studied by XRD (Figure 5), showing crystalline regions located at 22.6°, 16.5° and 14.5° corresponding to the (1ī0), (110) and (200) diffraction planes as reported in the literature (Jozala et al., 2015; Reiniati et al., 2017).

### 3.3 Static fermentation under different conditions

**FIGURE 6:**
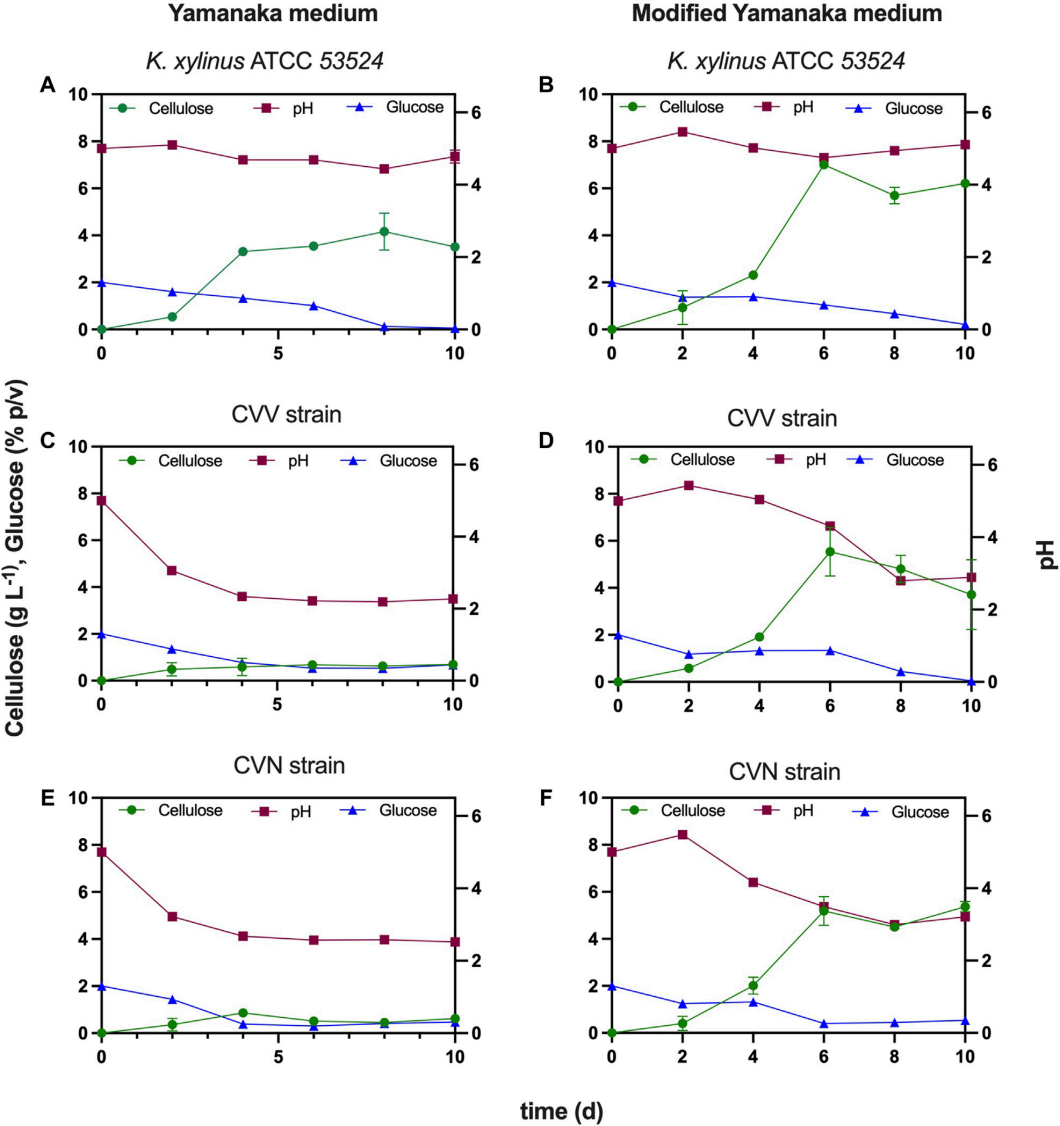
Comparison of BNC production using the isolated strains and the ATCC strain under different culture media for 10 days. Error bars are represented by the deviation standard of two experiments. **(A)** Reference strain (*K. xylinus* ATCC 53524) cultivated in Yamanaka medium, **(B)** Reference strain (*K. xylinus* ATCC 53524) cultivated in modified Yamanaka medium, **(C)** Isolated CVV cultivated in Yamanaka medium, **(D)** Isolated CVV cultivated in modified Yamanaka medium, **(E)** Isolated CVN cultivated in Yamanaka medium and **(F)** Isolated CVN cultivated in modified Yamanaka medium. Main left y-axis indicates BNC yield in g L^−1^ and glucose concentration in % p/v, while main y-right axis and x-axis indicate pH and time in days (d), respectively (for all graphs).

Static fermentation of *K. xylinus* ATCC 53524 did not lead to a significant change in the pH of the medium, which decreased from 5.0 to 4.78 after 10 days (Figure 6A). Cellulose production reached 3.3 g L^−1^ after day 4, slightly increasing to 3.5 g L^−1^ at day 10. Glucose, on the other hand, gradually decreased from day 0 (2%) and was almost completely consumed at day 8 (0.13%). However, the behavior of both isolated strains was characterized by showing at the second day a pronounced pH drops from 5 to near 3.2 (strain CVV) and to 3.06 (strain CVN), remaining stable around 2.5 (strain CVV) and 2.2 (strain CVN) during the following days (Figures 6C,E). In addition, glucose consumption decreased from 2% to 1.4% (strain CVV) and to 1.3% (strain CVN) at day 2, while at day 4 its consumption stopped completely at around 0.4% (strain CVV) and 0.6% (strain CVN). The production of BNC reached concentrations of 0.7 (strain CVV) and 0.6 g L^−1^ (strain CVN) after 10 days of fermentation. These values are significantly lower (≈80%) than those obtained with the ATCC strain, which results from the different metabolisms between the bacterial species. The low pH is well-established to be the result of metabolic by-products (e.g., gluconic acid), which has detrimental effects on the consumption rate of the carbon source, the growth of bacterial cells and over the production of cellulose (Zhong et al., 2013). In addition, pH reduction in the fermentation broth is known to impact the activity of key enzymes associated with the BNC biosynthetic pathway (Li et al., 2012).

It is possible to observe in Figure 6 that BNC production drastically increased for all bacterial strains upon incorporating citrate buffer in the Yamanaka medium (3.74 g L^−1^ of sodium citrate and 1.35 g L^−1^ of citric acid) (Figures 6B,D,F). The maximum yields obtained for CVV, CVN and *K. xylinus* ATCC 53524 were 5.5 g L^−1^ (day 6), 5.4 g L^−1^ (day 10) and 7.0 g L^−1^ (day 6), respectively. From the observation at the macroscopic level (Figure 3) this increment is evident, showing thicker and bigger films when the citrate-buffered media was used after 10 days of cultivation.

During fermentation with CVV strain, the pH of the culture media reached a value of 4 at day 6, however; at day 8 it went down to 2.8 and then stayed stationary until day 10. Glucose was completely consumed during the 10 days, while cellulose reached 4–5 g L^−1^ at days 6–10, which is 8 times higher than the yield obtained without including citrate buffer in the culture medium (Figure 6D). A similar trend was observed in the fermentation of the CVN strain, as the pH decreased to 3.5 by day 6 (obtaining a BNC yield of 5.2 g L^−1^) and then maintained invariable until day 10, when the pH reached 3.2 (Figure 6F).

However, unlike the isolated strains, *K. xylinus* ATCC 53524 was characterized by keeping the pH constant over a culture time of 10 days. In addition, BNC production increased to around 7.0 g L^−1^ when modified Yamanaka medium was employed, compared with a yield of 3.5 g L^−1^ under standard conditions (Figure 6B). This increment is also visible in Figure 3, since a thicker and more homogeneous pellicle is observed when using citrate-buffered medium after 10 days of fermentation. As pH is a key operational parameter affecting the BNC productivity, developing simple and effective ways to deal with acidification of the medium becomes paramount to achieve BNC production in a cost-effective manner and to help develop static fermentation.

As presented in Table 1, the two novel strains (CVV and CVN) and *K. xylinus* ATCC 53524 that were grown in citrate-buffered Yamanaka medium reached yields and productivities on the top 50%.

**TABLE 1:** Top-performing bacterial nanocellulose yields and productivities obtained under static fermentation and different pH conditions.

Strain	Medium	Time (d)	Yield (g L^−1^)	Productivity (g L^−1^d^−1^) Calculated from article data	References
*K. hansenii*	HS—1.5% Glucose and 2.5% Corn steep liquor pH 5-6	10	9.63	0.963	Costa et al. (2017)
*Leifsonia soli*	1% Maltose	7	5.97	0.853	Rastogi and Banerjee (2020)
	0.8% Soy whey				
	0.8% Calcium chloride pH 6.5				
*K. xylinus. (Formerly known as Acetobacter xylinum)*	Nicotine-removed tobacco waste extracts	First stage:7	First stage: 2.56	First stage: 0.366	Ye et al. (2019)
	First stage pH 6.5	Second stage 8–16	Second stage: 5.2	Second stage: 0.325	
	Second stage adjusting pH to 6.5 at day 7				
*K. xylinus* KTH 5655 (*formerly known as K. aceti*)	HS - 2% Glucose pH 5	9	10.39	1.154	Chen et al. (2017)
*K. xylinus*	YPD - 6% Glucose	8	7.23	0.904	Kuo et al. (2016)
	Acetate buffer ionic strength of 100 mM pH 4.75				
*K. medellensis (acid resistant strain)*	HS - 2% Glucose pH 3.5	8	4.5	0.563	Castro et al. (2012)
*K. hansenii*	HS - 2% glucose pH 5, buffered with	9	1) 5.16	1) 0.573	Li et al. (2021)
	1) phosphate buffer, ionic strength of 90 mM		2) 4.63	2) 0.514	
	2) Phthalate buffer, ionic strength of 73 mM				
*K. xylinus (UVC- mutations)*	BCEL-GC01%–5% Glucose pH 6	4	14.0	3.500	Lee et al. (2023)
1) *Komagateibacter CVV strain*	Yamanaka—2% Glucose Citrate buffer ionic strength 22 mM pH. 5	6	1) 5.5 2) 5.1 (5.4–10days) 3) 7.0	1) 0.917	This study
2) *Komagataeibacter CVN strain*				2) 0.850	
*3*) *K. xylinus ATCC 53524*				3) 1.167	

It is worth noting that *K. xylinus* KTH 5655 can produce large amounts of cellulose during long-term fermentation, even though typically the highest BNC production period of *Komagataeibacter* strains tend to occur between 2 and 5 days after fermentation starts (Masaoka et al., 1993; Son et al., 2001).

*Komagataeibacter* spp. have been described to achieve high but variable BNC yields (namely, *K. europaeus*, *K. hansenii*, *K. rhaeticus*, *K. medellinensis*, *K. melomenusus*, and *K. xylinus*), which metabolize different carbon sources at pH values fluctuating between neutral to slightly acidic values (Kesh and Sameshima, 2005). Accordingly, previous works have determined preferred pH values of the fermentation media around 4.5 to 7.5 for different BNC-producing strains (Son et al., 2001; Wang et al., 2018). However, an unusual acid-resistant strain (*Komagataeibacter* medellensis) was characterized with a maximum BNC production of 4.5 g L^−1^ achieved under acidic medium (pH 3.5) (Castro et al., 2012).

Sodium citrate is a triprotic organic acid with excellent stability and strong buffering properties, which can be used to regulate the pH in microbial cultures typically over a neutral pH range, since its pKa values are 3.13 (pK_a1_), 4.76 (pK_a2_) and 6.40 (pK_a3_). For example, a recent work demonstrated the effectivity of citrate buffer to stabilize the pH of fermentation broth (in the range 5.5–7.5) and to improve biohydrogen production performance using a photosynthetic bacterium (Guo et al., 2022). However, it is noteworthy that pK_a2_ of citrate lies within an optimal BNC production range that potentially makes it very suitable for buffering the pH drop caused by the generation acid by-products during fermentation. Despite this observation, only a few studies have addressed the use of citrate buffer in the context of BNC production (Zeng et al., 2011; Li et al., 2021). In particular, Li et al. (2021) investigated the effect of various pH buffers (phosphate, phthalate, acetate and citrate) on the BNC yield of *Komagataeibacter hansenii* strain ATCC 53582, using modified HS medium (with 2% glucose). The bacterium produced a good BNC yield (5.16 g L^−1^) in a phosphate buffer with low ionic strength; however; the substantial reduction in pH during fermentation (from 5.0 to 3.5) accounted for the inability of the buffer to neutralize the excess hydrogen ions. To stabilize the pH, the ionic strength was enhanced from 90 mM to 600 mM, but under these conditions the bacteria lost their ability to synthesize cellulose. Likewise, the synthesis of BNC was totally inhibited because of the high ionic strengths of HS medium containing citrate and acetate buffers (600 and 200 mM, respectively). The acetate buffer was assessed in another study to improve BNC production by *K. xylinus* in modified HS media. BNC produced after 8 days cultivation was 2.98 g L^−1^, while the yield obtained in non-buffered media was only 1.23 g L^−1^, showing a decrease in the pH until ≤3.5 (initial pH of 4.75, 5.50, and 6.00) (Li et al., 2021).

In summary, the improvements achieved in BNC yield and productivities may be directly related with the buffering effect of citrate, which prevented pH to drop below 4.0 (until day 6) and thus avoiding inhibition of cell growing. Both strains (CVV and CVN) showed long-term cellulose synthesis ability (6–10 days), reaching good BNC-yields (5.4-5-5 g L^−1^) and productivities (∼0.9 g L^−1^ d^−1^).

It is worth noting that citrate itself can act as a carbon source in the fermentation processes and the cometabolism with glucose is well-documented in the literature for lactic acid bacteria (LAB). However, this is an unstable trait since the ability to metabolize citrate is dependent on the presence of a plasmid-encoded enzyme that plays a crucial role in the uptake of citrate into the cell (citrate permease) (Eicher et al., 2024). Subsequent conversion of citrate into acetate and oxaloacetate via citrate lyase-oxaloacetate decarboxylase supplies intracellular pools of pyruvate that can then be reoriented toward different metabolic pathways (Eicher et al., 2024). Accordingly, citrate cometabolism has been associated with an enhancement of the growth of *Leuconostoc spp*., in contrast to other LAB species such as *Lactobacillus casei* and *Lactobacillus plantarum* and *Enterococcus faecium*, where citrate does not affect the cellular growth (Frederik et al., 2006). Likewise, Ricciardi et al. (2022) showed in a recent work that citrate boosted the growth rate of *Leuconostoc mesenteroides* only in anaerobiosis; while its supplementation decreased the μ_max_ under respiratory conditions.

**FIGURE 7:**
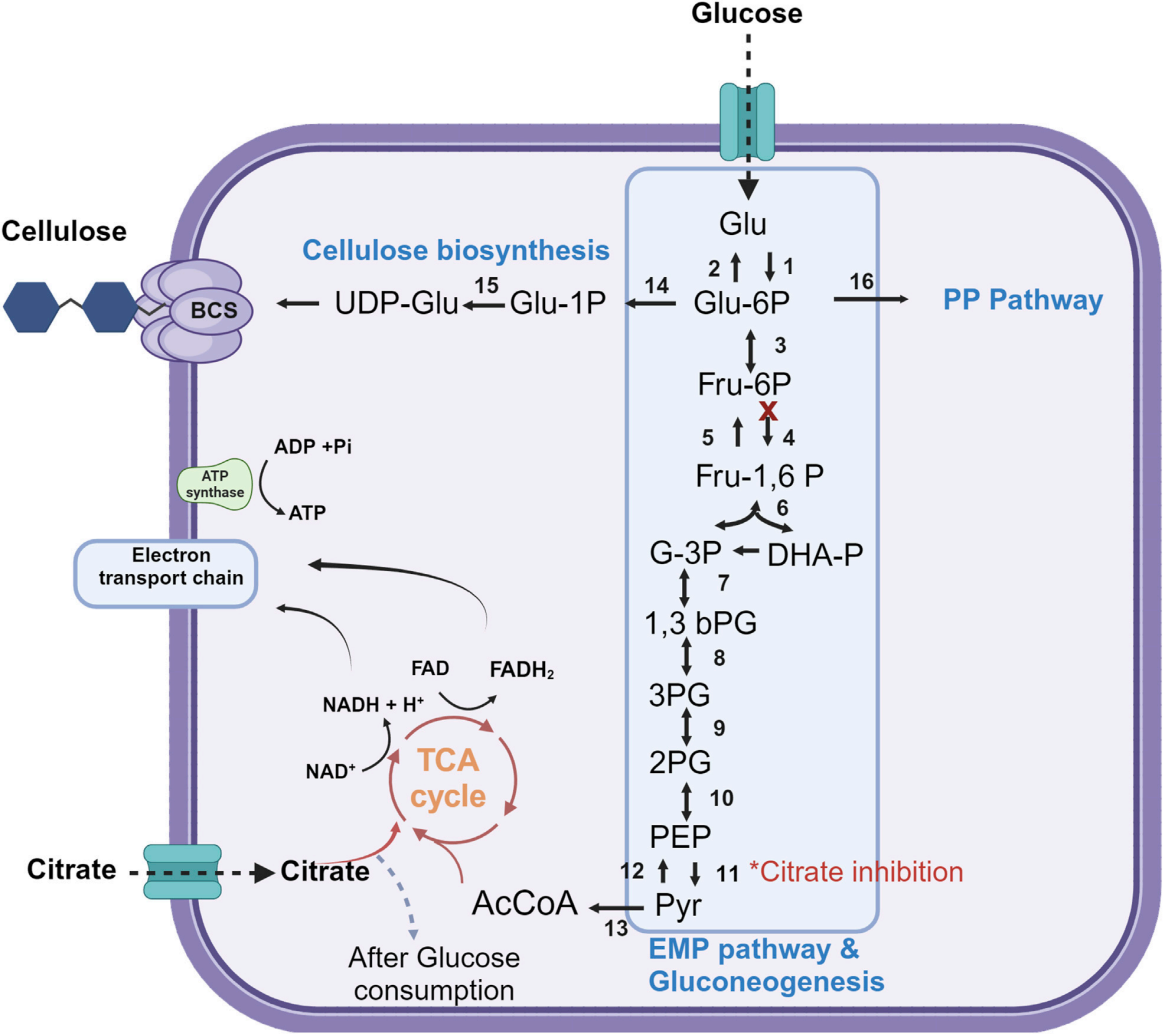
Overview of the key metabolic pathways for BNC biosynthesis. Glu: glucose, Glu-1P: glucose-1phosphate, Glu 6-P: glucose 6-phosphate, UDP-Glu: Uridine diphosphate glucose, Fru: fructose, Fru-6-P: fructose 6-phosphate, Fru-1,6-P: fructose 1,6-biphosphate, DHA-P: dihydroxyacetone phosphate, 3PG: 3 phosphoglycerate, 1,3 bPG: 1,3-bisphosphoglycerate, 2PG: 2 phosphoglycerate, 3PG: 3 phosphoglycerate, PEP: phosphoenolpyruvate, Pyr: pyruvate, AcCoA: acetyl coenzyme A, TCA cycle: Tricarboxylic acid cycle, PP Pathway: Pentose Phosphate pathway, BCS: Bacterial Cellulose Synthase Complex (1) hexokinase, (2) glucose 6 phophatase, (3) phosphoglucose isomerase, (4) phosphofructokinase, (5) fructose 1,6 bisphosphatase, (6) aldolase, (7) glyceraldehyde 3 phosphate dehydrogenase, (8) phosphoglycerate kinase, (9) phosphoglycerate mutase, (10) enolase, (11) pyruvate kinase, (12) pyruvate carboxylase and PEP carboxy kinase, (13) pyruvate dehydrogenase, (14) phosphoglucomutase, (15) UDP glucose pyro phosphorylase, (16) glucose 6 phosphate dehydrogenase. This figure is based on Jacek et al. (2019), Manan et al. (2022) and Li et al. (2012). Created with BioRender.com.

The genetic ability for citrate assimilation has been characterized in *Acetobacter* species (Illeghems et al., 2016), however; the catabolic pathways are poorly described. *K. xylinus* (formerly named *Acetobacter xylinum*) may use citrate as carbon source in the presence of glucose, being metabolized only after glucose is depleted (Geyer et al., 1994; Jonas and Farah, 1998). In other study, when citrate was used as the carbon source it yielded only a 20% production rate compared to glucose (both carbon sources at 0.5% p/v) (Masaoka et al., 1993; Jonas and Farah, 1998). The biosynthesis of BNC is connected to various metabolic pathways (Figure 7), including the pentose-phosphate (PP) pathway, the Embden–Meyerhof–Parnas (EMP) pathway, the Krebs cycle (TCA), and gluconeogenesis (Jacek et al., 2019). In *Komagataeibacter* spp the phosphofructokinase gene of the EMP pathway is either absent or the enzyme exhibits low activity, indicating an incomplete metabolic pathway for pyruvate synthesis (Nascimento et al., 2021; Manan et al., 2022). Citrate was shown to inhibit the activity of pyruvate kinase (PK) in a study performed by Li et al. (2012) with *Novacetimonas hansenii,* a bacterial species belonging to the *Acetobacteraceae* family. This enzyme serves as a crucial regulatory enzyme in the EMP pathway, where it catalyzes the transference of a phosphate group from phosphoenolpyruvate (PEP) to ADP and yields one molecule of pyruvate and one molecule of ATP (Li et al., 2012). Therefore, the subsequent accumulation of PEP is expected to increase the carbon flux toward BNC via the gluconeogenesis pathway. In this work, the authors studied the effect of supplementing a glucose basal medium (glucose 5%, yeast extract 0.5%, peptone 0.5%, Na_2_HPO_4_ 0.2%, K_2_HPO_4_ 0.1%, citric acid 0.1%, and MgSO_4_ 0.025%) with 2% citrate and/or 2% ethanol to study BNC production in static culture of *Novacetimonas hansenii*. Ethanol supplementation enabled 2.8 g/L^−1^ BNC in 7 days (a 142% yield increase), accounting for a substantial increase in ATP concentration upon conversion of ethanol to acetate by alcohol dehydrogenase. High ATP levels are well-known to stimulate BNC production through negatively regulating the enzymes controlling the glycolytic pathway (phosphofructokinase and pyruvate kinase) and the TCA cycle (pyruvate dehydrogenase and isocitrate dehydrogenase). Interestingly, a 7% additional increase in BNC production was determined by supplementing both citrate and ethanol, however; the effect of citrate alone was not investigated.

Using the reference strain *K. xylinus* ATCC 53524 we obtained a 100% increase in BNC production when grown in modified Yamanaka media. As pH remained constant throughout the fermentation using non-buffered Yamanaka medium, this enhancement could be explained in part from the modulator role of citrate, by depressing glycolysis and favoring BNC synthesis through the gluconeogenesis pathway, and in a lesser degree by assimilation of citrate as an additional carbon source. This minor effect is also supported by a previous study where citrate only yielded 20% of the BNC compared to an equal amount of glucose as the carbon source (Masaoka et al., 1993). On the other hand, this increment is notably lower than that observed in BNC yield of both isolated strains, where pH buffering caused a more drastic effect on the performance. The inclusion of sodium citrate and citric acid in the Yamanaka medium contributes about 15% of the total cost of the chemical reagents (see Supplementary Material), which can be considered a rather marginal increase given the substantial enhancement in BNC yield. Interestingly, this buffer system could be potentially useful to regulate pH and optimize fermentation costs in complex media that incorporate agroindustrial by-products. For example, depending on the origin and composition of the by-product the pH may increase or fall outside the optimal range during BNC production (Heydorn et al., 2023).

In particular, the strain *K. xylinus* KTH 5655 has been previously characterized by producing high levels of BNC at day 9 of the cultivation time (10.39 g L^−1^), in HS medium at pH 5.0. Despite several *Komagataeibacter* strains have been investigated to maximize BNC production under static culture, the comparison with a common basis becomes challenging due to heterogeneity of media components and culture conditions (see Table 1). Accordingly, the fermentative production of BNC is strongly dependent on factors including carbon and nitrogen sources, temperature, pH and reactor type (Chawla et al., 2009). On another arena, an UVC-induced mutant strain of *K. xylinus* ATCC 53524 (named LYP25) was generated to improve BNC fermentation performance, using a rich growth medium consisting of 10 g L^−1^ yeast extract, 20 g L^−1^ peptone and 5% glucose (YPD medium) for seed culture and fermentation experiments. The mutant LYP25 achieved a maximum BNC production of 14.0 g L^−1^ at day 4 of the fermentation, exceeding by 41% the production of the wild-type strain (yield of 9.9 g L^−1^), with an initial glucose level of 50 g L^−1^ that was fully consumed by day 6. Herein, using the same control strain (*K. xylinus* ATCC 53524) we achieved a maximum yield of 4.2 g L^−1^ that likely accounts for the lower initial glucose level (2%). Likewise, the strain *K. hansenii* UCP1619 was evaluated for production of BNC pellicles using alternative media formulated with different carbon and nitrogen sources, such as yeast extract, peptone, and corn steep liquor (CSL). The top resulting yield (9.63 g L^−1^) was obtained in 1.5% glucose supplemented with 2.5% CSL.

These results are of interest in the light of improving the culture media to address pH drop and thus to increase the feasibility of scaling-up the bioprocess. The standard technology for BNC production is static fermentation, which produces a highly homogeneous supramolecular BNC structure with higher crystallinity, stronger tensile strength and denser network structure, among other superior properties (Gao et al., 2020). Static reactors are simpler and have no need of sophisticated instruments, being thus an ideal method for manufacturing flat BNC for commercial applications (Reshmy et al., 2021). However, a main issue that may hinder the industrial application of these systems is their low productivity associated with features such as larger cultivation areas, longer fermentation times and limited control over process variables such as pH and oxygen supply (El-Gendi et al., 2022). The adoption of agitated culture has been proposed as a solution to these problems, holding potential for large scale production of BNC thanks to its higher mass transfer rate and improved aeration conditions. However, this technology faces challenges such as complex bacterial strain instability, non-Newtonian behavior during BNC mixing and a large shear force. In addition, BNC generated by agitated cultures exhibits low degree of polymerization, low crystallinity index and poor mechanical characteristics (Kouda et al., 1996; Kouda et al., 1997). Interestingly, BNC productivities determined in this work for CVV (0.917 g L^−1^ d^−1^) and CVN (0.850 g L^−1^ d^−1^) strains compare favorably with previous results from studies based on static and agitated culture systems. For instance, Chen et al. (2019) compared the performance of both types of production strategies, finding conditions of low speed (100 rpm) under which shaking cultivation reached a superior productivity of BNC (1.14 g L^−1^ d^−1^) than that obtained by static culture (0.55 g L^−1^ d^−1^). Given this, we provide evidence for novel bacterial strains together with a simple yet effective buffering strategy to address the pH problem and to allow top-performing BNC production in static culture.

## 4 Conclusion

The production of bacterial nanocellulose was investigated using two novel strains isolated from artisanal apple vinegar (CVV and CVN strains) and compared with the reference *K. xylinus* ATCC 53524 strain. The new strains were identified as *Komagateibacter* spp. and produced pellicles that were correctly characterized as nanocellulose by SEM, FTIR and XRD techniques. Importantly, modifications on Yamanaka medium by adding citrate buffering capacity (3.74 g L^−1^ of sodium citrate and 1.35 g L^−1^ of citric acid) lead to top-performing BNC production. The highest BNC yields were obtained at day 6 of cultivation for CVV (5.5 g L^−1^) and CVN (5.4 g L^−1^) strains, while productivities in 6 days of fermentation achieved top-performing levels (0.850–0.917 g L^−1^ d^−1^). We hypothesized this enhancement results from a combination of factors instead of one single cause, which mainly include the buffering properties of the modified medium to maintain favorable pH conditions during the fermentation; with additional contributions from the regulator roles of citrate that can increase carbon flux towards BNC production via gluconeogenesis pathway; and providing an additional carbon source after glucose consumption. The relative effect of these factors is expected to be the subject of further studies.

The increase in costs derived from the incorporation of the buffer reagents into Yamanaka medium represents about 15% of the overall costs of the fermentation medium, which is arguably a modest effect in the light of the yield boost achieved in BNC production. In addition, this buffer system holds potential to be studied in complex media that incorporate agroindustrial by-products to regulate pH with a low-cost impact. These results provide valuable insight into novel bacterial strains and strategies to improve static fermentation and the feasibility of scaling-up these systems for industrial applications.

## Data Availability

The datasets presented in this study can be found in online repositories. The names of the repository/repositories and accession number(s) can be found in the article/Supplementary Material.

## References

[B1] Anguluri K. La China S. Brugnoli M. Cassanelli S. Gullo M. (2022). Better under stress: improving bacterial cellulose production by Komagataeibacter xylinus K2G30 (UMCC 2756) using adaptive laboratory evolution. Front. Microbiol. 13, 994097. 10.3389/fmicb.2022.994097 36312960 PMC9605694

[B2] Bennich T. Belyazid S. (2017). The route to sustainability—prospects and challenges of the bio-based economy. Sustainability 9, 887. 10.3390/su9060887

[B3] Blanco Parte F. G. Santoso S. P. Chou C.-C. Verma V. Wang H.-T. Ismadji S. (2020). Current progress on the production, modification, and applications of bacterial cellulose. Crit. Rev. Biotechnol. 40, 397–414. 10.1080/07388551.2020.1713721 31937141

[B4] Brandão P. R. Crespo M. T. B. Nascimento F. X. (2022). Phylogenomic and comparative analyses support the reclassification of several Komagataeibacter species as novel members of the Novacetimonas gen. nov. and bring new insights into the evolution of cellulose synthase genes. Int. J. Syst. Evol. Microbiol. 72. 10.1099/ijsem.0.005252 35175916

[B5] Brugnoli M. Robotti F. La China S. Anguluri K. Haghighi H. Bottan S. (2021). Assessing effectiveness of Komagataeibacter strains for producing surface-microstructured cellulose via guided assembly-based biolithography. Sci. Rep. 11, 19311. 10.1038/s41598-021-98705-2 34588564 PMC8481549

[B6] Cacicedo M. L. Castro M. C. Servetas I. Bosnea L. Boura K. Tsafrakidou P. (2016). Progress in bacterial cellulose matrices for biotechnological applications. Bioresour. Technol. 213, 172–180. 10.1016/j.biortech.2016.02.071 26927233

[B7] Castro C. Zuluaga R. Álvarez C. Putaux J.-L. Caro G. Rojas O. J. (2012). Bacterial cellulose produced by a new acid-resistant strain of Gluconacetobacter genus. Carbohydr. Polym. 89, 1033–1037. 10.1016/j.carbpol.2012.03.045 24750910

[B8] Chawla P. Bajaj I. B. Survase S. A. Singhal R. S. (2009). Microbial cellulose: fermentative production and applications. Food Technol. Biotechnol. 47, 107–124. 10.1155/2015/280784

[B9] Chen G. Wu G. Chen L. Wang W. Hong F. F. Jönsson L. J. (2019). Performance of nanocellulose-producing bacterial strains in static and agitated cultures with different starting pH. Carbohydr. Polym. 215, 280–288. 10.1016/j.carbpol.2019.03.080 30981355

[B10] Chen S.-Q. Mikkelsen D. Lopez-Sanchez P. Wang D. Martinez-Sanz M. Gilbert E. P. (2017). Characterisation of bacterial cellulose from diverse Komagataeibacter strains and their application to construct plant cell wall analogues. Cellulose 24, 1211–1226. 10.1007/s10570-017-1203-3

[B11] Costa A. F. S. Almeida F. C. G. Vinhas G. M. Sarubbo L. A. (2017). Production of bacterial cellulose by gluconacetobacter hansenii using corn steep liquor as nutrient sources. Front. Microbiol. 8, 2027. 10.3389/fmicb.2017.02027 29089941 PMC5651021

[B12] Dirisu G. Rosenzweig J. Lambert E. Oduah A. (2017). pH effect and pH changes during biocellulose production by gluconacetobacter xylinus in moringa oleifera tea-sugar medium. JAMB 7, 1–7. 10.9734/JAMB/2017/38440

[B13] Eicher C. Coulon J. Favier M. Alexandre H. Reguant C. Grandvalet C. (2024). Citrate metabolism in lactic acid bacteria: is there a beneficial effect for Oenococcus oeni in wine? Front. Microbiol. 14, 1283220. 10.3389/fmicb.2023.1283220 38249489 PMC10798043

[B14] El-Gendi H. Taha T. H. Ray J. B. Saleh A. K. (2022). Recent advances in bacterial cellulose: a low-cost effective production media, optimization strategies and applications. Cellulose 29, 7495–7533. 10.1007/s10570-022-04697-1

[B15] Esa F. Tasirin S. M. Rahman N. A. (2014). Overview of bacterial cellulose production and application. Agric. Agric. Sci. Procedia 2, 113–119. 10.1016/j.aaspro.2014.11.017

[B16] Fernandes I. de A. A. Pedro A. C. Ribeiro V. R. Bortolini D. G. Ozaki M. S. C. (2020). Bacterial cellulose: from production optimization to new applications. Int. J. Biol. Macromol. 164, 2598–2611. 10.1016/j.ijbiomac.2020.07.255 32750475

[B17] Frederik V. Veerle G. Effie T. Luc De V. (2006). Cometabolism of citrate and glucose by Enterococcus faecium FAIR-E 198 in the absence of cellular growth. Appl. Environ. Microbiol. 72, 319–326. 10.1128/AEM.72.1.319-326.2006 16391060 PMC1352224

[B18] Gao H.-L. Zhao R. Cui C. Zhu Y.-B. Chen S.-M. Pan Z. (2020). Bioinspired hierarchical helical nanocomposite macrofibers based on bacterial cellulose nanofibers. Natl. Sci. Rev. 7, 73–83. 10.1093/nsr/nwz077 34692019 PMC8289019

[B19] Geyer U. Klemm D. Schmauder H.-P. (1994). Kinetics of the utilization of different C sources and the cellulose formation by Acetobacter xylinum. Acta Biotechnol. 14, 261–266. 10.1002/abio.370140308

[B20] Guindon S. Gascuel O. (2003). A simple, fast, and accurate algorithm to estimate large phylogenies by maximum likelihood. Syst. Biol. 52, 696–704. 10.1080/10635150390235520 14530136

[B21] Gullo M. Sola A. Zanichelli G. Montorsi M. Messori M. Giudici P. (2017). Increased production of bacterial cellulose as starting point for scaled-up applications. Appl. Microbiol. Biotechnol. 101, 8115–8127. 10.1007/s00253-017-8539-3 28965208

[B22] Guo S. Lu C. Wang K. Wang J. Zhang Z. Liu H. (2022). Effect of citrate buffer on hydrogen production by photosynthetic bacteria. Bioresour. Technol. 347, 126636. 10.1016/j.biortech.2021.126636 34971780

[B23] Guzman-Puyol S. Benítez J. J. Heredia-Guerrero J. A. (2022). Sustainable bio-based polymers: towards a circular bioeconomy. Polymers 14, 22. 10.3390/polym14010022 PMC8747281 35012045

[B71] Hestrin S. Schramm M. (1954). Synthesis of cellulose by *Acetobacter xylinum*. II. Preparation of freeze-dried cells capable of polymerizing glucose to cellulose. Biochem. J. 58, 345–352. 10.1042/bj0580345 13208601 PMC1269899

[B24] Heydorn R. L. Lammers D. Gottschling M. Dohnt K. (2023). Effect of food industry by-products on bacterial cellulose production and its structural properties. Cellulose 30, 4159–4179. 10.1007/s10570-023-05097-9

[B25] Illeghems K. Pelicaen R. De Vuyst L. Weckx S. (2016). Assessment of the contribution of cocoa-derived strains of Acetobacter ghanensis and Acetobacter senegalensis to the cocoa bean fermentation process through a genomic approach. Food Microbiol. 58, 68–78. 10.1016/j.fm.2016.03.013 27217361

[B26] Islam M. U. Ullah M. W. Khan S. Shah N. Park J. K. (2017). Strategies for cost-effective and enhanced production of bacterial cellulose. Int. J. Biol. Macromol. 102, 1166–1173. 10.1016/j.ijbiomac.2017.04.110 28487196

[B72] Jacek P. Dourado F. Gama M. Bielecki S. (2019). Molecular aspects of bacterial nanocellulose biosynthesis. Microb. Biotechnol. 12, 633–649. 10.1111/1751-7915.13386 30883026 PMC6559022

[B27] Jonas R. Farah L. F. (1998). Production and application of microbial cellulose. Polym. Degrad. Stab. 59, 101–106. 10.1016/S0141-3910(97)00197-3

[B28] Jozala A. F. Pértile R. A. N. dos Santos C. A. de Carvalho Santos-Ebinuma V. Seckler M. M. Gama F. M. (2015). Bacterial cellulose production by Gluconacetobacter xylinus by employing alternative culture media. Appl. Microbiol. Biotechnol. 99, 1181–1190. 10.1007/s00253-014-6232-3 25472434

[B29] Kesh S. M. A. S. Sameshima K. (2005). Evaluation of different carbon sources for bacterial cellulose production. Afr. J. Biotechnol. 4, 478–482. 10.4314/ajb.v4i6.15125

[B30] Klemm D. Schumann D. Udhardt U. Marsch S. (2001). Bacterial synthesized cellulose — artificial blood vessels for microsurgery. Prog. Polym. Sci. 26, 1561–1603. 10.1016/S0079-6700(01)00021-1

[B31] Kouda T. Yano H. Yoshinaga F. (1997). Effect of agitator configuration on bacterial cellulose productivity in aerated and agitated culture. J. Ferment. Bioeng. 83, 371–376. 10.1016/S0922-338X(97)80144-4

[B32] Kouda T. Yano H. Yoshinaga F. Kaminoyama M. Kamiwano M. (1996). Characterization of non-Newtonian behavior during mixing of bacterial cellulose in a bioreactor. J. Ferment. Bioeng. 82, 382–386. 10.1016/0922-338X(96)89155-0

[B33] Kuo C. H. Chen J. H. Liou B. K. Lee C. K. (2016). Utilization of acetate buffer to improve bacterial cellulose production by Gluconacetobacter xylinus. Food Hydrocoll. 53, 98–103. 10.1016/j.foodhyd.2014.12.034

[B34] La China S. De Vero L. Anguluri K. Brugnoli M. Mamlouk D. Gullo M. (2021). Kombucha tea as a reservoir of cellulose producing bacteria: assessing diversity among komagataeibacter isolates. Appl. Sci. 11, 1595. 10.3390/app11041595

[B35] Lahiri D. Nag M. Dutta B. Dey A. Sarkar T. Pati S. (2021). Bacterial cellulose: production, characterization, and application as antimicrobial agent. Int. J. Mol. Sci. 22, 12984. 10.3390/ijms222312984 34884787 PMC8657668

[B36] Lee J. Lee K. H. Kim S. Son H. Chun Y. Park C. (2023). Improved production of bacterial cellulose using Gluconacetobacter sp. LYP25, a strain developed in UVC mutagenesis with limited viability conditions. Int. J. Biol. Macromol. 232, 123230. 10.1016/j.ijbiomac.2023.123230 36641021

[B37] Lee K.-Y. Buldum G. Mantalaris A. Bismarck A. (2014). More than meets the eye in bacterial cellulose: biosynthesis, bioprocessing, and applications in advanced fiber composites. Macromol. Biosci. 14, 10–32. 10.1002/mabi.201300298 23897676

[B38] Li Y. Tian C. Tian H. Zhang J. He X. Ping W. (2012). Improvement of bacterial cellulose production by manipulating the metabolic pathways in which ethanol and sodium citrate involved. Appl. Microbiol. Biotechnol. 96, 1479–1487. 10.1007/s00253-012-4242-6 22782249

[B39] Li Z. Chen S. Q. Cao X. Li L. Zhu J. Yu H. (2021). Effect of pH buffer and carbon metabolism on the yield and mechanical properties of bacterial cellulose produced by *Komagataeibacter hansenii* ATCC 53582. J. Microbiol. Biotechnol. 31, 429–438. 10.4014/jmb.2010.10054 33323677 PMC9705897

[B40] Liu X. Wu M. Wang M. Hu Q. Liu J. Duan Y. (2022). Direct synthesis of photosensitizable bacterial cellulose as engineered living material for skin wound repair. Adv. Mater. 34, 2109010. 10.1002/adma.202109010 35076119

[B41] Manan S. Ullah M. W. Ul-Islam M. Shi Z. Gauthier M. Yang G. (2022). Bacterial cellulose: molecular regulation of biosynthesis, supramolecular assembly, and tailored structural and functional properties. Prog. Mater. Sci. 129, 100972. 10.1016/j.pmatsci.2022.100972

[B42] Masaoka S. Ohe T. Sakota N. (1993). Production of cellulose from glucose by Acetobacter xylinum. J. Ferment. Bioeng. 75, 18–22. 10.1016/0922-338X(93)90171-4

[B43] Mohite B. V. Patil S. V. (2014). A novel biomaterial: bacterial cellulose and its new era applications. Biotechnol. Appl. Biochem. 61, 101–110. 10.1002/bab.1148 24033726

[B44] Nascimento F. X. Torres C. A. V. Freitas F. Reis M. A. M. Crespo M. T. B. (2021). Functional and genomic characterization of Komagataeibacter uvaceti FXV3, a multiple stress resistant bacterium producing increased levels of cellulose. Biotechnol. Rep. 30, e00606. 10.1016/j.btre.2021.e00606 PMC7970039 33747802

[B45] Neelima S. Sreejith S. Shajahan S. Raj A. Vidya L. Aparna V. M. (2023). Highly crystalline bacterial cellulose production by Novacetimonas hansenii strain isolated from rotten fruit. Mater. Lett. 333, 133622. 10.1016/j.matlet.2022.133622

[B46] Núñez D. Cáceres R. Ide W. Varaprasad K. Oyarzún P. (2020). An ecofriendly nanocomposite of bacterial cellulose and hydroxyapatite efficiently removes lead from water. Int. J. Biol. Macromol. 165, 2711–2720. 10.1016/j.ijbiomac.2020.10.055 33069824

[B47] Paradis E. Schliep K. (2019). Ape 5.0: an environment for modern phylogenetics and evolutionary analyses in R. Bioinformatics 35, 526–528. 10.1093/bioinformatics/bty633 30016406

[B48] Petersen N. Gatenholm P. (2011). Bacterial cellulose-based materials and medical devices: current state and perspectives. Appl. Microbiol. Biotechnol. 91, 1277–1286. 10.1007/s00253-011-3432-y 21744133

[B49] Picheth G. F. Pirich C. L. Sierakowski M. R. Woehl M. A. Sakakibara C. N. de Souza C. F. (2017). Bacterial cellulose in biomedical applications: a review. Int. J. Biol. Macromol. 104, 97–106. 10.1016/j.ijbiomac.2017.05.171 28587970

[B50] Portela R. Leal C. R. Almeida P. L. Sobral R. G. (2019). Bacterial cellulose: a versatile biopolymer for wound dressing applications. Microb. Biotechnol. 12, 586–610. 10.1111/1751-7915.13392 30838788 PMC6559198

[B51] Posada D. Crandall K. A. (1998). MODELTEST: testing the model of DNA substitution. Bioinformatics 14, 817–818. 10.1093/bioinformatics/14.9.817 9918953

[B52] Puri V. P. (1984). Effect of crystallinity and degree of polymerization of cellulose on enzymatic saccharification. Biotechnol. Bioeng. 26, 1219–1222. 10.1002/bit.260261010 18551639

[B53] Qi Y. Guo Y. Liza A. A. Yang G. Sipponen M. H. Guo J. (2023). Nanocellulose: a review on preparation routes and applications in functional materials. Cellulose 30, 4115–4147. 10.1007/s10570-023-05169-w

[B54] Rastogi A. Banerjee R. (2020). Statistical optimization of bacterial cellulose production by Leifsonia soli and its physico-chemical characterization. Process Biochem. 91, 297–302. 10.1016/j.procbio.2019.12.021

[B55] Reiniati I. Hrymak A. N. Margaritis A. (2017). Kinetics of cell growth and crystalline nanocellulose production by Komagataeibacter xylinus. Biochem. Eng. J. 127, 21–31. 10.1016/j.bej.2017.07.007

[B56] Reshmy R. Eapen P. Deepa T. Araving M. Raveendran S. Parameswaran B. (2021). Bacterial nanocellulose: engineering, production, and applications. Bioengineered 12, 11463–11483. 10.1080/21655979.2021.2009753 34818969 PMC8810168

[B57] Ricciardi A. Storti L. V. Giavalisco M. Parente E. Zotta T. (2022). The effect of respiration, pH, and citrate Co-metabolism on the growth, metabolite production and enzymatic activities of Leuconostoc mesenteroides subsp. cremoris E30. Foods 11, 535. 10.3390/foods11040535 35206012 PMC8871477

[B58] Ruka D. R. Simon G. P. Dean K. M. (2012). Altering the growth conditions of Gluconacetobacter xylinus to maximize the yield of bacterial cellulose. Carbohydr. Polym. 89, 613–622. 10.1016/j.carbpol.2012.03.059 24750766

[B59] Ryngajłło M. Jędrzejczak-Krzepkowska M. Kubiak K. Ludwicka K. Bielecki S. (2020). Towards control of cellulose biosynthesis by Komagataeibacter using systems-level and strain engineering strategies: current progress and perspectives. Appl. Microbiol. Biotechnol. 104, 6565–6585. 10.1007/s00253-020-10671-3 32529377 PMC7347698

[B60] Salari M. Sowti Khiabani M. Rezaei Mokarram R. Ghanbarzadeh B. Samadi Kafil H. (2019). Preparation and characterization of cellulose nanocrystals from bacterial cellulose produced in sugar beet molasses and cheese whey media. Int. J. Biol. Macromol. 122, 280–288. 10.1016/j.ijbiomac.2018.10.136 30342939

[B61] Son H.-J. Heo M. S. Kim Y. G. Lee S. J. (2001). Optimization of fermentation conditions for the production of bacterial cellulose by a newly isolated Acetobacter. Biotechnol. Appl. Biochem. 33, 1–5. 10.1042/BA20000065 11171030

[B62] Thomas B. Raj M. C. B A. K. H R. M. Joy J. Moores A. (2018). Nanocellulose, a versatile green platform: from biosources to materials and their applications. Chem. Rev. 118, 11575–11625. 10.1021/acs.chemrev.7b00627 30403346

[B63] Torgbo S. Sukyai P. (2018). Bacterial cellulose-based scaffold materials for bone tissue engineering. Appl. Mater. Today 11, 34–49. 10.1016/j.apmt.2018.01.004

[B64] Wahid F. Hu X. H. Chu L. Q. Jia S. R. Xie Y. Y. Zhong C. (2019). Development of bacterial cellulose/chitosan based semi-interpenetrating hydrogels with improved mechanical and antibacterial properties. Int. J. Biol. Macromol. 122, 380–387. 10.1016/j.ijbiomac.2018.10.105 30342151

[B65] Wang S. S. Han Y. H. Chen J. L. Zhang D. C. Shi X. X. Ye Y. X. (2018). Insights into bacterial cellulose biosynthesis from different carbon sources and the associated biochemical transformation pathways in komagataeibacter sp. W1. Polymers 10, 963. 10.3390/polym10090963 30960888 PMC6403882

[B66] Yarza P. Yilmaz P. Pruesse E. Glöckner F. O. Ludwig W. Schleifer K. H. (2014). Uniting the classification of cultured and uncultured bacteria and archaea using 16S rRNA gene sequences. Nat. Rev. Microbiol. 12, 635–645. 10.1038/nrmicro3330 25118885

[B67] Ye J. Zheng S. Zhang Z. Yang F. Ma K. Feng Y. (2019). Bacterial cellulose production by Acetobacter xylinum ATCC 23767 using tobacco waste extract as culture medium. Bioresour. Technol. 274, 518–524. 10.1016/j.biortech.2018.12.028 30553964

[B68] Zeng X. Liu J. Chen J. Wang Q. Li Z. Wang H. (2011). Screening of the common culture conditions affecting crystallinity of bacterial cellulose. J. Industrial Microbiol. Biotechnol. 38, 1993–1999. 10.1007/s10295-011-0989-5 21630052

[B69] Zhang M. Chen S. Sheng N. Wang B. Wu Z. Liang Q. (2021). Spinning continuous high-strength bacterial cellulose hydrogel fibers for multifunctional bioelectronic interfaces. J. Mater. Chem. A 9, 12574–12583. 10.1039/D1TA01606G

[B70] Zhong C. Zhang G. C. Liu M. Zheng X. T. Han P. P. Jia S. R. (2013). Metabolic flux analysis of Gluconacetobacter xylinus for bacterial cellulose production. Appl. Microbiol. Biotechnol. 97, 6189–6199. 10.1007/s00253-013-4908-8 23640364

