# Supplementary material for: Citrate-buffered Yamanaka medium allows to produce high-yield bacterial nanocellulose in static culture using Komagataeibacter strains isolated from apple cider vinegar
Source: Front Bioeng Biotechnol. 2024 May 15;12:1375984. doi: 10.3389/fbioe.2024.1375984 (PMC11133569; doi:10.3389/fbioe.2024.1375984)
Supplement: Supplementary file 1 [file Table1.DOCX]

**Citrate-buffered Yamanaka medium allows to produce high-yield bacterial nanocellulose in static culture using *Komagataeibacter* strains isolated from apple cider vinegar.**

Dariela Núñez ^1, 2*^, Patricio Oyarzún ^3*^, Rodrigo Cáceres^1^, Elizabeth Elgueta^1, 2^, Maribet Gamboa^1, 2^

^1^ Departamento de Química Ambiental, Facultad de Ciencias, Universidad Católica de la Santísima Concepción, Concepción, Chile

^2^ Centro de Investigación en Biodiversidad y Ambientes Sustentables (CIBAS), Universidad Católica de la Santísima Concepción, Concepción, Chile

^3^ Facultad de Ingeniería, Arquitectura y Diseño, Universidad San Sebastián, Lientur 1457, Concepción 4080871, Chile.

* **Corresponding authors:**

*Dariela Núñez,* [*dnunez@ucsc.cl*](mailto:dnunez@ucsc.cl)*,*

*Patricio Oyarzún,* [*patricio.oyarzún@uss.cl*](mailto:patricio.oyarzún@uss.cl)*,*

**Supplementary material**

Table S1: Cost determination of the Yamanaka and citrate-buffered medium.

| Yamanaka medium | Composition (g L^-1^) | Cost  (USD Kg^-1^) | Cost (USD L^-1^) |
| --- | --- | --- | --- |
| Glucose | 20 | 21 | 0,42 |
| Ammonium sulfate | 5 | 22,6 | 0,113 |
| Yeast extract | 5 | 140 | 0,7 |
| K_2_HPO_4_ | 3 | 30 | 0,09 |
| Magnesium sulfate | 0,05 | 25 | 0,00125 |
| Sodium citrate | 3,74 | 45 | 0,1683 |
| Citric acid | 1,35 | 23 | 0,03105 |

The costs were determined for lab-scale production from an existing supplier (Winkler Ltda.) and the component quotes were taken in march 2024

Table S2: Evaluation of the citrate buffer cost on the total cost of Yamanaka medium.

| Yamanaka medium | Total cost (USD L^-1^) |
| --- | --- |
| with buffer | 1,5 |
| without buffer | 1,3 |
| % cost increase | 15,1 |
